# Comparison of different approaches to combined spinal epidural anesthesia (CSEA) under the guidance of ultrasound in cesarean delivery of obese patients: a randomized controlled trial

**DOI:** 10.1186/s40001-021-00577-9

**Published:** 2021-09-15

**Authors:** Yilu Zhou, Wei Chen, Shuangqiong Zhou, Yiyi Tao, Zhendong Xu, Zhiqiang Liu

**Affiliations:** 1 Department of Anesthesiology, Shanghai First Maternity and Infant Hospital, School of Medicine, Tongji University, 200092 Shanghai, China; 2 Deparment of Operation Room, Shanghai First Maternity and Infant Hospital, School of Medicine, Tongji University, 200092 Shanghai, China; 3 Department of Anesthesiology, Shanghai First Maternity and Infant Hospital, School of Medicine, Tongji University, 200092 Shanghai, China

**Keywords:** Different approaches, Combined spinal epidural anesthesia, Ultrasound, Cesarean delivery, Obese patients

## Abstract

**Background:**

Combined spinal epidural anesthesia (CSEA) is commonly performed in cesarean deliveries. However, it is difficult to perform in obese parturients because of positioning challenges. The aim of this study was to compare the effect of different approaches to CSEA under the guidance of ultrasound.

**Methods:**

One hundred obese patients (BMI ≥ 30 kg/m^2^) who underwent elective cesarean section were randomly enrolled. Patients were assigned to a median approach group and a paramedian approach group randomly. Clinical characteristics were compared between groups. First-attempt success rate, the median positioning time and total operation time, ultrasonic predicted anesthesia puncture depth, actual puncture depth, anesthesia adverse reactions, complications after anesthesia, and patients’ satisfaction with the epidural puncture were recorded.

**Results:**

The first-attempt success rate was significantly different between the two groups [92% (46/50) vs. 76% (38/50), *P*  =  0.029]. The median positioning time and total operation time in the paramedian approach group were higher than those in the median approach group (227.7 s vs. 201.6 s, *P * =  0.037; 251.3 s vs. 247.4 s, *P*  =  0.145). The incidence of postanesthesia complications in the paramedian approach group was significantly lower than that in the median approach group (2% vs. 12%, *P*  =  0.026), and patient satisfaction was higher in the paramedian approach group than in the median approach group (*P*  =  0.032).

**Conclusion:**

The ultrasound-guided paramedian approach for CSEA is time-consuming, but it can effectively improve the success rate of the first puncture, reduce the incidence of anesthesia-related adverse reactions, and improve patient satisfaction.

*Trial registration*: This study was registered with the Chinese Clinical Trial Registry (ChiCTR1900024722) on July 24, 2019

## Background

Combined spinal epidural anesthesia (CSEA) has the advantages of quick onset, good effect and controllable action time. It has been widely used in clinical practice and became the preferred anesthesia method for cesarean section [1]. However, failure of intraspinal anesthesia puncture caused by a large abdominal circumference, a non-ideal anesthesia position, obesity and tissue edema, as well as anesthesia-related complications, such as nerve injury, unsatisfactory anesthetic effect and postpartum lumbago [2]. Therefore, it is particularly important to improve the success rate of CSEA for pregnant women, especially for obese patients.

Ultrasound technology has the advantages of easy operation and has been widely valued in clinical practice. Previous studies have shown that ultrasound imaging of the spine has the ability to assist in locating the epidural space [3, 4] and can also be used to measure the distance from the skin to the epidural space to predict the penetration depth of the puncture needle to avoid the puncture of the dura mater caused by a too-deep puncture. Some studies have shown that although there was a certain difference between the ultrasonic prediction of puncture depth and the actual operation of epidural puncture depth, there is still a good correlation between them [5]. This provides some guidance for anesthesiologists for conducting ultrasound-guided epidural puncture. Epidural puncture by anesthesiologists under the guidance of ultrasound has become one of the hot topics of clinical anesthesia and pain treatment research [6]. Studies have found that there may be no significant difference in the timing between traditional intraspinal puncture by experienced anesthesiologists and ultrasound-guided epidural puncture [7–9], which does not reflect the application advantage of ultrasound in epidural puncture. However, ultrasound localization is helpful for epidural puncture in patients with difficult surface localization or abnormal anatomical markers. A recent study found that ultrasound positioning can be used for intraspinal anesthesia in cesarean section of obese pregnant women [10]. The first puncture success rate in the ultrasound group was significantly improved, the number of punctures was significantly reduced, the incidence of postoperative low back pain of parturients was reduced, and the safety of anesthesia was increased. These findings are consistent with the conclusions of other researchers [11].

However, some studies have found that the incidence of postpartum low back pain was high in overweight or obese women after spinal anesthesia [12], and the incidence of postpartum low back degeneration was high in obese women [13]. There are two puncture approaches for combined spinal epidural anesthesia: median approach puncture and paramedian approach puncture. Early studies have noted that the success rate of paramedian approach puncture was higher than that of median approach puncture and that it is associated with fewer complications and postoperative complications [14]. To date, we found that all the relevant studies on intraspinal anesthesia have used the median approach puncture by ultrasound; however, it is still unknown whether the paramedian approach under ultrasound can improve the success rate of puncture and reduce complications when compared with the median approach. Therefore, we conducted a randomized controlled study to compare the difference between median approach group and paramedian approach group. In this study, we hypothesized that the paramedian approach under ultrasound guidance can improve the success rate of the first puncture in obesity parturient.

## Methods

### Materials and methods

**Fig. 1:**
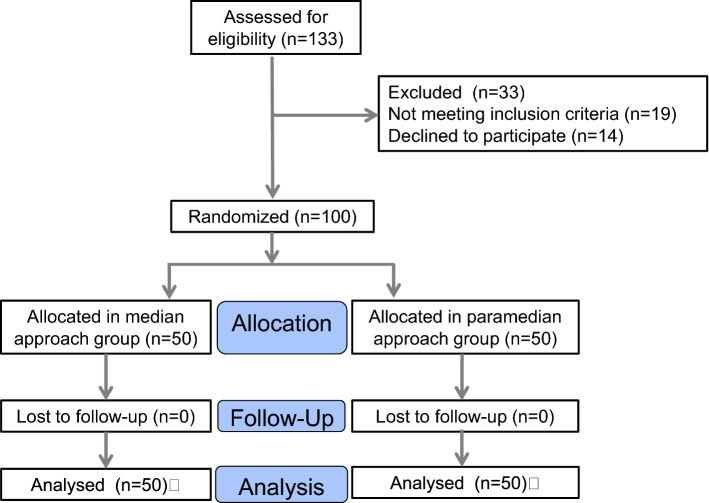
Flowchart of the subject recruitment process

The study was conducted in accordance with the Declaration of Helsinki (as revised in 2013). This study was approved by the Ethics Committee of Shanghai First Maternity and Infant Hospital and was registered with the Chinese Clinical Trial Registry (ChiCTR1900024722). All patients consented to the data being used for research when receiving treatment. A total of 100 obese pregnant women who underwent elective cesarean section in Shanghai First Maternity and Infant Hospital from August 2019 to March 2020 were selected. The inclusion criteria were as follows: (1) age  ≥  18 years, (2) normal singleton pregnancy, (3) gestational age  ≥  37 weeks, (4) body mass index (BMI)  ≥  30 kg/m^2^ (based on weight measured the day before delivery). The exclusion criteria were as follows: rejection of spinal anesthesia; twins; a history of spinal deformity or spinal surgery; contraindications to spinal anesthesia (infection of the puncture site, coagulation dysfunction, allergy to local anesthesia, insufficient blood volume or abnormal spinal anatomy) and emergency cesarean section. Combined spinal epidural anesthesia was performed on all patients, and the L3–4 or L2–3 interspace was selected. Participants will be randomized to median approach group or paramedian approach group on a 1:1 basis using a computer-generated randomization sequence. According to the different puncture approaches, 50 patients were divided into median approach group and paramedian approach group. No sedation was provided before or during anesthesia (Fig. 1). One anesthesiologist with more than 3 years of clinical experience in combined spinal epidural anesthesia was selected as the operator. Ultrasound was performed by a single researcher trained in the technique, and more than 150 ultrasound-guided spinal block experiences were performed.

### Anesthesia care

**Fig. 2:**
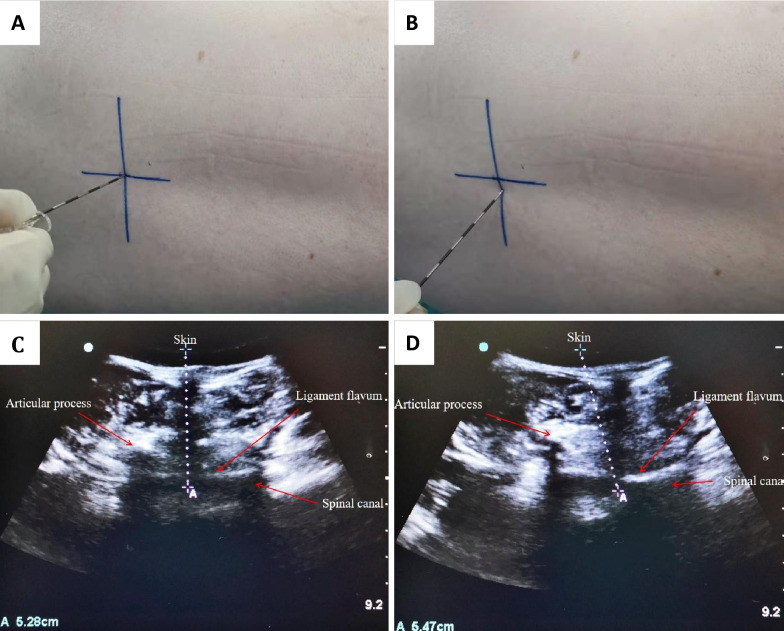
**A** Location of median approach on the skin. **B** Location of paramedian approach on the skin. **C** The distance from the skin to the epidural space guided by ultrasound in the median approach group. **D** The distance from the skin to the epidural space guided by ultrasound in the paramedian approach group

The patient was placed in the right-side position with the arms embracing the knees and the back arched. A Sonosite convex array probe (MTurbo ultrasound system, Fujifilm SonoSite, Inc., Bothell, Washington 98,021 USA) was used for ultrasonic scanning. The ultrasonic probe was placed at the middle level of the sacrococcygeal region, and a scan was performed horizontally and moved to the lumbosacral region. The L3 vertebra, L4 vertebra and L3–4 interspace were identified, the skin was marked, and the probe was turned to the horizontal position while keeping it centered. Then, the position of the L3 vertebra was determined and marked, and the intersection of the longitudinal and transverse lines at the L3–4 interspace was determined as the puncture point for the median approach. In longitudinal sagittal ultrasound imaging, it is necessary to identify the L3 and L4 articular processes and the ligamentum flavum in the middle of the articular processes and to measure the distance between the skin and ligamentum flavum to predict the puncture depth (Fig. 2A, C). In the paramedian approach group, the L3–4 interspace was selected under ultrasound, and the puncture point was 1.5 cm below this area in the median vertical paracentesis. And the distance between the skin and the ligamentum flavum was measured by ultrasound at the puncture point to predict the depth of anesthesia puncture (Fig. 2B, D).

After the skin was marked, full aseptic precautions were exercised, then 1% lidocaine was injected into the skin for local anesthesia, and it was confirmed that the 16G epidural puncture needle had entered into the epidural space (negative pressure method). The patient was instructed to keep still, and the subarachnoid cavity was entered through the epidural needle cavity with a 25G lumbar anesthesia needle. After cerebrospinal fluid appeared in the lumbar puncture needle, the needle tip was pointed toward the patient's head, and 0.5% ropivacaine 2–3 mL was injected at rate of approximately 0.1 mL/s. After injection, the spinal needle was pulled out, an epidural catheter was placed, and the patient was moved to left supine position. In the paramedian approach group, the angle between the needle and the skin was 75°, and other steps were as the same as in the median approach group.

The L3–4 interspace was the first choice for puncture, the L2–3 interspace was used for the follow-up attempt. A maximum of 3 skin puncture attempts (needle withdrawn from the skin and then readvanced) were allowed for one interspace and a maximum of 5 needle passes (needle withdrawn and readvanced without complete withdrawal from the skin) were allowed for each skin puncture attempt. If dural puncture was unsuccessful after attempts at the L2–3 interspace, the operator was allowed to use other means to perform anesthesia, including changing the operator or anesthesia mode. Successful spinal anesthesia was defined by a bilateral T4 block five minutes after injection. The incidence of hypotension (mean blood pressure below 90 mmHg or systolic pressure reduction of  >  25% from the initial value) was recorded. Other complications, such as bloody tap or paresthesia, were also recorded by an independent observer blinded to the group allocation. A blinded attending anesthesiologist recorded all the outcomes.

### Measurement

The clinical data of the patients, including age, gestational age, height, weight, ASA grade, BMI and duration of surgery were recorded. The primary outcome was the rate of successful dural puncture on the first attempt. Secondary outcomes were the location time (from the time of the operator placed the ultrasonic probe on the back of the patient to the end of positioning), total operation time (the time from disinfection to the time when the patient changed to supine position), adverse reactions during puncture (incidence of nerve stimulation, epidural catheter bleeding), complications after anesthesia (incidence of low back pain) and patients’ satisfaction after surgery.

## Statistical analysis

Statistical analysis was performed using SPSS Version 22.0 (IBM, Armonk, NY). Normally distributed outcome data were summarized as the mean (standard deviation) and compared between groups using independent measures *t* test. Categorical data were analyzed using the *χ*^2^ test. Fisher’s exact test was used for subgroup analyses for subgroups. Reported *P* values were not corrected for multiple testing. *P*  <  0.05 were considered statistically significant.

## Results

A total of 133 women were recruited for the study from August 2019 to March 2020, after exclusion there were 100 patients in analysis. There were 50 patients in the paramedian approach group and 50 patients in the median approach group. No patients were excluded due to loss of data or failed follow-up (Fig. 1). Demographic characteristics were summarized in (Table 1).

**Table 1:** Baseline of patient characteristics between the groups

	Median approach group (*n* = 50)	Paramedian approach group (*n* = 50)
Age (mean, standard deviations), year	32.53 (8.53)	32.02 (10.02)
Gestational age (mean, standard deviations), days	273.6 (6.56)	274.1 (6.13)
Height (mean, standard deviations), cm	160.57 (11.43)	161.36 (12.64)
Weight (mean, standard deviations), kg	83.21 (15.79)	84.47 (19.53)
ASA grade	–	–
Grade I	8	10
Grade II	42	40
Duration of surgery (mean, standard deviations), min	36.66 (23.33)	33.57 (24.43)
BMI (mean, standard deviations), kg/m^2^	32.37 (4.13)	32.41 (6.09)
Obesity grading	–	–
30 ≤ BMI ≤ 34.9	38 (76%)	39 (78%)
34.9 ≤ BMI ≤ 39.9	7 (14%)	5 (10%)
40 ≤ BMI	5 (10%)	6 (12%)

Data are present with mean (standard deviations)

*ASA* American Society of Anesthesiologists, *BMI* body mass index

Procedure-related data between the two groups are shown in Table 2. The success rate of the first attempt in the paramedian approach group was significantly higher [92% (46/50) vs. 76% (38/50), *P*  =  0.029] than that in the median approach group. Two groups of patients achieved bilateral T4 block after successful anesthesia, and there were no cases in which the mode of anesthesia was changed. There was no significant difference between the two groups in the total operation time of puncture (251.3 s vs. 247.4 s, *P*  =  0.145), but the location time of the paramedian approach group was significantly longer than that of the median approach group (227.7 s vs. 201.6 s, *P*  =  0.037). In addition, there were some differences in anesthesia-related adverse reactions between the two groups. Compared with that in the median approach group, lower nerve stimulation occurred during anesthesia puncture (1/50 vs. 2/50, *P*  =  0.742) in the paramedian approach group. There was a significant difference in epidural catheter bleeding (2/50 vs. 3/50, *P*  =  0.686), and the incidence of postoperative low back pain between the two groups (1/50 vs. 6/50, *P*  =  0.026). Patients in the paramedian approach group had higher satisfaction than those in the median approach group (*P*  =  0.032).

**Table 2:** Comparisons of procedure-related data between groups

	Median approach group (*n* = 50)	Paramedian approach group (*n* = 50)	*P *value
First-attempt success rate	38 (76%)	46 (92%)	0.029*
Location time (median, interquartile), seconds	201.6 (169.3–219.5)	227.7 (183.7–231.8)	0.037*
Total operation time (median, interquartile), seconds	247.4 (225.3–272.8)	251.3 (228.7–276.8)	0.145
Anesthesia adverse reactions			
Nerve stimulation	2	1	0.742
Epidural catheter bleeding	3	2	0.686
Low back pain	6	1	0.026*
Satisfaction			
Very satisfied	15	35	0.032*
Satisfied	32	14	
Dissatisfied	3	1	

*mean P < 0.05

Table 3 shows the data of the ultrasonic predicted anesthesia puncture depth and actual puncture depth in the two groups. There was no significant difference in the actual puncture depth between the two groups (in the median approach group, *P*  =  0.927; in the paracentral approach group, *P*  =  0.726).

**Table 3:** Comparison of the ultrasonic prediction anesthesia puncture depth and the actual puncture depth in the two groups

	Ultrasonic prediction puncture depth	Actual puncture depth	*P *value
Median approach group (mean, standard deviations)	5.63 (1.52)	5.67 (1.67)	0.927
Paramedian approach group (mean, standard deviations)	5.66 (1.82)	5.81 (1.74)	0.726

Data are present with mean (standard deviations)

## Discussion

In this randomized controlled study, we found that the first puncture success rate in the paramedian approach group was 92%, which was significantly higher than the 76% in the median approach group. In the paramedian approach, the superior and interspinous ligaments were avoided so that the epidural space was entered directly from the ligamentum flavum. In addition, based on the analysis of the anatomical structure of the spine, the paramedian approach is not limited by the inclination of the spinous process and the bone structure. When entering the epidural space, the end of the puncture needle is more inclined to form an angle on the side of the head, the cerebrospinal fluid returns smoothly after the insertion of the lumbar anesthesia needle, and it is easier to place the epidural catheter after the completion of the lumbar anesthesia. Therefore, the first puncture success rate was higher in the paramedian approach group than in the median approach group. Some studies have confirmed that the puncture interspace of the paramedian approach is wider than that of median approach, which reduces the difficulty of puncture, avoids repeated puncture and increases the success rate of puncture [15].

At the same time, the controversy over the use of ultrasound to assess the distance between the skin and the epidural space should be considered. In theory, the actual puncture depth is deeper than that predicted by ultrasound. Our study found that the actual depth of extradural puncture was deeper than that predicted by ultrasound, but there was no significant difference between groups. The reason may be that when the operator evaluates the success of epidural puncture, the needle insertion is stopped immediately when negative pressure is felt during the puncture to avoid placing the puncture needle too deeply, especially for anesthesiologists with more puncture experience. There was no significant difference between the predicted puncture depth and the actual puncture depth in the median approach group, which suggested that ultrasound could effectively predict the epidural puncture depth in different approaches of CSEA. However, it should be pointed out that the actual puncture depth of both groups of data was deeper than that predicted by ultrasound. Considering the possible influence of fat thickness or tissue edema on the backs of obese pregnant women, our study compressed the maternal skin to avoid its influence when ultrasound placed the puncture point.

In this study, a low-frequency ultrasound probe was used to accurately determine the best puncture point of anesthesia in the median approach group. The selection of puncture point in the paramedian approach group was based on the lateral paracentesis of 1.5 cm at the median approach puncture point, and on this basis, ultrasound was used to predict the puncture depth. Therefore, it was found that the location time of the paramedian group was higher than that of the median group. However, there was no significant difference in the total operation time between the two groups. Considering the first puncture success rate of the two groups was high, the number of attempts was small, and the skilled operation of anesthesiologists had a certain relationship.

Anesthesia safety is also an important factor in this study. Among the 100 obese pregnant women included in this study, there were adverse effects of anesthesia in both groups, such as epidural catheter bleeding, nerve stimulation signs and the occurrence of low back pain after anesthesia, and there were significant differences between the two groups in the occurrence of low back pain. We considered that the paramedian approach group could avoid the supraspinous ligament and part of the interspinous ligament and allow entrance into the epidural space through the ligamentum flavum in the process of puncture. The analysis shows that the main reason for the difference in the incidence of low back pain between the two groups is the difference in ligament injury caused by the dural puncture needle. There was no significant difference in nerve stimulation between the two groups. It is worth mentioning that there were no cases of unexpected dural puncture in the neither of the groups, further suggesting the advantage of ultrasound in obese women. There was a higher degree of satisfaction in the paramedian approach group than in the median approach group.

Previous studies have found that there is no significant difference in the success rate of traditional intraspinal puncture when ultrasound is used or not in non-obese pregnant patient [16], which does not reflect the application advantages of ultrasound in epidural puncture. Therefore, this study is more valuable for the application of ultrasound-guided epidural puncture in obese cesarean section women. In addition, it should be pointed out that the technology of ultrasound intervention in epidural puncture can be divided into prepuncture positioning and real-time guiding operation, but the real-time guiding requires aseptic treatment of the probe, the operation process is complex, and the advantage is not obvious compared with the prepuncture ultrasound positioning; additionally, in the operation process, elimination of air/liquid resistance is still used to determine whether the tip of the needle reaches the epidural cavity[17]. Therefore, in this study, we chose to place the epidural catheter before ultrasound-guided puncture rather than under real-time ultrasound-guided puncture. Of course, there are some limitations in our research. Firstly, although we used the same anesthesiologist with much experience in using spinal ultrasound, the results are still controversial. Secondly, we had a relatively small number of cases and fewer positive results. More samples are needed for further study. Thirdly, we are lacking for a group without use of ultrasound in this study and highly consider enrolling this group in a future study.

## Conclusion

Our conclusion is that the ultrasound-guided paramedian approach puncture for CSEA under the guidance of ultrasound in cesarean delivery obese patients is more time-consuming, but it can effectively improve the success rate of the first puncture, reduce the incidence of anesthesia-related adverse reactions, and improve patient satisfaction.

## Data Availability

Not applicable.

## Abbreviations

CSEA: Combined spinal epidural anesthesia
BMI: Body mass index
ASA: American Society of Anesthesiologists classification
